# From Humoral Theory to Performant Risk Stratification in Kidney Transplantation

**DOI:** 10.1155/2017/5201098

**Published:** 2017-01-02

**Authors:** C. Lefaucheur, D. Viglietti, M. Mangiola, A. Loupy, A. Zeevi

**Affiliations:** ^1^Paris Translational Research Center for Organ Transplantation, INSERM, UMR-S970, Paris, France; ^2^Kidney Transplant Department, Saint-Louis Hospital, Assistance Publique-Hôpitaux de Paris, Paris, France; ^3^Department of Transplant Pathology, University of Pittsburgh Medical Center, Pittsburgh, PA, USA; ^4^Kidney Transplant Department, Necker Hospital, Assistance Publique-Hôpitaux de Paris, Paris, France

## Abstract

The purpose of the present review is to describe how we improve the model for risk stratification of transplant outcomes in kidney transplantation by incorporating the novel insights of donor-specific anti-HLA antibody (DSA) characteristics. The detection of anti-HLA DSA is widely used for the assessment of pre- and posttransplant risks of rejection and allograft loss; however, not all anti-HLA DSA carry the same risk for transplant outcomes. These antibodies have been shown to cause a wide spectrum of effects on allografts, ranging from the absence of injury to indolent or full-blown acute antibody-mediated rejection. Consequently, the presence of circulating anti-HLA DSA does not provide a sufficient level of accuracy for the risk stratification of allograft outcomes. Enhancing the predictive performance of anti-HLA DSA is currently one of the most pressing unmet needs for facilitating individualized treatment choices that may improve outcomes. Recent advancements in the assessment of anti-HLA DSA properties, including their strength, complement-binding capacity, and IgG subclass composition, significantly improved the risk stratification model to predict allograft injury and failure. Although risk stratification based on anti-HLA DSA properties appears promising, further specific studies that address immunological risk stratification in large and unselected populations are required to define the benefits and cost-effectiveness of such comprehensive assessment prior to clinical implementation.

## 1. Introduction

Circulating anti-donor-specific HLA antibodies (anti-HLA DSA) were recognized in hyperacute rejection in 1969 [1]; however, it took more than 40 years for the transplant community to consider the presence of anti-HLA DSA as the main reason for allograft rejection and long-term failure [2, 3]. There is mounting evidence both experimental and clinical in support of Dr. Terasaki's prediction as outlined in “the humoral theory of transplantation” [4, 5]. Furthermore, the transplant community has recognized circulating anti-HLA DSA detected prior to or after transplantation as one of the most informative biomarkers for predicting worse allograft outcome [6].

Although the detection of anti-HLA DSA is widely used in clinical practice for the assessment of pre- and posttransplant risks of rejection and allograft loss, it has become indisputable that not all anti-HLA DSA carry the same risk for transplant outcomes [7]. These antibodies have been shown to cause a wide spectrum of effects on allografts, ranging from the absence of injury to indolent or full-blown acute antibody-mediated rejection (ABMR) [8, 9]. Consequently, the presence of circulating anti-HLA DSA does not provide a sufficient level of accuracy for the risk stratification of allograft outcome. Enhancing the predictive performance of anti-HLA DSA is currently one of the most pressing unmet needs for facilitating individualized treatment choices that may improve outcomes [7].

Over the last decade, studies have been focused on defining how the level of circulating anti-HLA DSA may explain the substantial phenotypic variability in allograft injury. First, anti-HLA DSA strength (mean fluorescent intensity [MFI] as defined by Luminex single antigen bead testing [SAB]) has been associated with antibody-mediated allograft injury and risk of allograft loss. Currently, the strength of anti-HLA DSA defined by MFI is used in allocation policies and immunological monitoring after transplantation. However, recent data have demonstrated that the level of HLA antibodies cannot be determined by SAB testing of undiluted sera and serial dilutions are required to assess the titer of the antibody [10]. In addition, a more comprehensive assessment of circulating anti-HLA DSA that includes their capacity to bind complement and their IgG subclass composition would also provide clinically relevant information with respect to the prediction of allograft injury and loss.

The purpose of the present review is to describe how we improve the model for risk stratification of transplant outcomes in kidney transplantation by incorporating the novel insights of anti-HLA DSA characteristics.

## 2. Contemporary Multidimensional Assessment of Circulating Donor-Specific Anti-HLA Antibodies

Introduction of multiplex-bead array assays has significantly improved the sensitivity and precision of circulating anti-HLA DSA detection. The benefits and limitations of the solid-phase assays using SAB have been captured in many reviews identifying potential problems that may impact test interpretation of antibody strength and patient management [7, 12]. For example, false positive results may be reported due to antibodies to denatured HLA molecules, or false weak or negative results may occur in the presence of intrinsic and extrinsic factors inhibiting the SAB assay [13]. It was elegantly demonstrated in two studies that the false low MFI in SAB assays, “prozone,” was caused by C1 complex formation that initiates classical complement activation culminating in dense C3b/d deposition, thus preventing secondary antibody binding [14, 15]. Furthermore, biologic confounding factors related to epitope-sharing may also impact the MFI values. Currently SABs may provide a semiquantitative measurement of antibody strength but are not approved for quantitative assessment of antibody level. Removing potential inhibitors in the sera with various treatment modalities has improved HLA antibody detection, but it did not address the potential oversaturation of the beads in the presence of high titer antibody. Tambur et al. demonstrated that serial dilution of sera pre-SAB testing provided a reliable measure of antibody strength over time and was informative for monitoring antibody levels pre- and postdesensitization protocols [10, 16].

Although the standard SAB assay has improved the sensitivity of HLA antibody testing, it does not discriminate between complement-binding IgG and noncomplement-binding subclasses [7]. Flow cytometry based detection of HLA antibody using FlowPRA beads was the first cell-independent assay to demonstrate complement activation in vitro [17]. Recently, two SAB assays have been developed to detect C1q- or C3d-binding antibodies [18–27]. The ability of HLA antibody to bind complement has been shown to depend on the composition of IgG subtypes: complement-binding IgG1 or IgG3 versus noncomplement-binding IgG2 and IgG4 subtypes [28]. However, we have shown in sensitized renal transplant recipients that merely the presence of complement-binding IgG subtype in the mixture was not enough to detect C1q-binding antibody [28]. Many studies attempted to show a strong correlation between strength of antibody (>8000 MFI) and C1q-binding reactivity [29]. The best correlation, however, was found between HLA antibody titer >1 : 16 or 1 : 32 and complement-binding ability [10, 30]. We have also compared the neat MFI, C1q reactivity, and IgG subtype level (MFI) in a group of sensitized renal transplant recipients [28]. For example, despite the strong total IgG SAB MFI (8000–11000), C1q reactivity was negative for anti A2, A68, A23, B13, DR12, and DR1; IgG subtypes for these specificities consisted of only low level IgG1 and/or IgG2 (Table 1). In contrast, HLA antibodies that consisted of a combination of multiple IgG subtypes were more often C1q-reactive, as long as one of the subtypes was IgG1 or IgG3 (anti-B53, DR10, DQ6, DQ7/DQA1*∗*05, DQ7/DQA1*∗*03, and DQB1*∗*05:01). Interestingly, anti-HLA-B53 was complement-binding even though it consisted of strong level IgG4 but in combination with IgG1, IgG2, and IgG3 whereas anti-HLA-B35 was not complement-binding; it consisted of similar strong level IgG4 in combination with low level IgG1 and IgG3 (Table 1). These few examples illustrate the complexity of complement-binding capacity of HLA antibody and considering the composition of the IgG subtypes and their level may be more informative to predict C1q reactivity rather than the neat MFI of SAB assay. Of note, none of the examples depicted in Table 1 were considered “prozone” since the total IgG MFI was >8000 with or without C1q binding. In contrast, in prozone the SAB MFI value for the HLA antibody is low while the C1q-SAB MFI is high [7, 10, 13, 30]. Removing the complement interference by DTT, heat, or EDTA treatment has improved the interpretation of the SAB assay; however, it did not address the limitations of SAB assay for determining the titer of DSA nor the composition of the IgG subtypes.

In summary, based on the current knowledge of SAB testing, to use a single MFI value to predict clinical outcomes is not sufficient. Comprehensive monitoring to facilitate risk assessment and patient-tailored management should incorporate an algorithm that addresses HLA antibody characteristics.

## 3. Circulating Donor-Specific Anti-HLA Antibodies for Risk Stratification in Organ Transplantation

**Figure 1:**
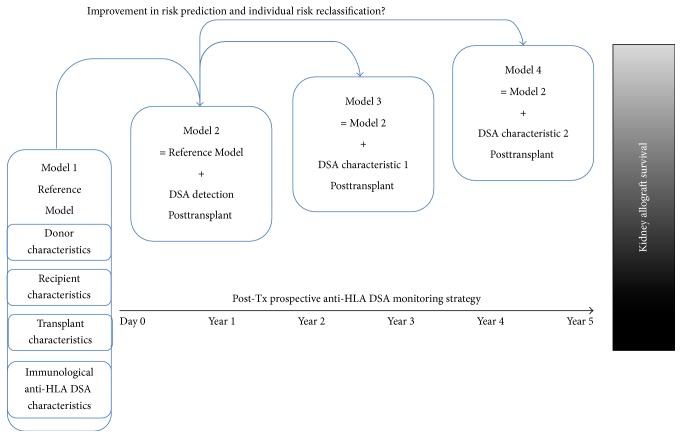
Prospective strategy of dynamic, incremental modeling to assess improvement in risk prediction of allograft loss based on circulating anti-HLA DSA monitoring and characterization. DSA, donor-specific antibody; HLA, human leucocyte antigen; Tx, transplant.

The present review was focused on prospective cohort studies that used hard endpoint (allograft loss) among observational studies that assessed the clinical value of anti-HLA DSA in order to provide the best level of evidence. To date, most studies in kidney transplantation have been limited to association analyses between the anti-HLA DSA and ABMR occurrence, allograft histological lesions, or allograft failure. Furthermore, the detection of anti-HLA DSA in an individual patient has not been shown to improve the accuracy of existing prediction model based on conventional risk factors [31]. In contrast, in other fields such as cancer or cardiovascular diseases, emerging biomarkers have made an important impact on risk prediction [32, 33]. A novel strategy using a dynamic integration of anti-HLA DSA and their characteristics should be addressed using dedicated metrics for discrimination and risk reclassification [34–36]. An illustration of such a strategy is provided in Figure 1.

### 3.1. The Value of Donor-Specific Anti-HLA Antibody Detection for Predicting Outcomes of Kidney Transplantation: Role of Systematic Monitoring

Short-term and long-term kidney allograft survival have been shown to be substantially worse among patients with pretransplant anti-HLA DSA detected by cell-based assays using complement-dependent cytotoxicity testing [1] or flow cytometry crossmatching [37], compared with both sensitized patients without anti-HLA DSA and nonsensitized patients. This observation remains valid even in patients with preexisting anti-HLA DSA detected only by solid-phase assays such as the SAB Luminex technique with a 1.98-fold increase in the risk of ABMR and a 1.76-fold increase in the risk of allograft failure [38]. Because of the detrimental effect of preexisting anti-HLA DSA on kidney allograft outcome it became important to include this factor in national, regional, and local allocation policies worldwide. These policies have implemented rules to prevent transplantation in the presence of preexisting anti-HLA DSA by defining acceptable and unacceptable mismatches and performing virtual crossmatching [39–41].

In the posttransplant setting, the development of de novo anti-HLA DSA has also been reported to dramatically increase the risk of ABMR and allograft loss. Wiebe et al. [42] found a 10-year allograft survival rate of 57% in patients with de novo anti-HLA HLA DSA compared to 96% in patients without de novo anti-HLA DSA. Recently, the relevance of a prospective strategy of systematic posttransplant anti-HLA DSA monitoring using SAB Luminex for the prediction of the risk of allograft loss was demonstrated at the population level [11]. In this study, the detection of posttransplant anti-HLA DSA improved the performance of a conventional model defined at the time of transplantation (which included donor age, donor serum creatinine, cold ischemia time, and anti-HLA DSA status at day 0) for predicting allograft loss (increase in c-statistic from 0.67 to 0.72) [11].

Importantly, the detrimental effects of posttransplant anti-HLA DSA can occur in the absence of initial allograft dysfunction, and 12 to 58% of sensitized recipients with preexisting or de novo anti-HLA DSA might develop subclinical forms of ABMR and have an increased risk of allograft loss [42–45]. This further emphasizes the need for anti-HLA DSA monitoring to identify patients who might be at risk for developing ABMR. However, the low positive predictive value of anti-HLA DSA for identifying subclinical ABMR [11, 42, 46] has required allograft biopsies to be performed when posttransplant anti-HLA-DSA are detected to accurately determine if subclinical ABMR is present. Recent advances for characterizing anti-HLA DSA have been implemented to improve their predictive performance by identifying harmful anti-HLA DSA that are responsible for allograft injury and failure.

### 3.2. The Strength of Donor-Specific Anti-HLA Antibodies for Predicting Outcomes of Kidney Transplantation

Currently, the assessment of circulating anti-HLA DSA strength is widely used by transplant centers worldwide to stratify the pre- and posttransplant risks for ABMR and allograft loss [7]. Anti-HLA DSA strength is commonly assessed by the MFI value provided by SAB tests or the mean channel shift provided by cell-based flow cytometry crossmatches [47]. Although determining anti-HLA DSA level by solid-phase assay was not approved by the US Food and Drug Administration as a quantitative measurement [48], studies have defined clinically relevant anti-HLA antibodies detected only by this assay. Several groups have demonstrated correlations between increased MFI/mean channel shift levels and increased incidences of ABMR and allograft loss [49, 50]. These studies may imply that additional clinically relevant information beyond the presence or absence of anti-HLA DSA may be derived by considering the numeric values reported by these assays. Higher strength defined by MFI of circulating anti-HLA DSA have also been correlated with increased microvascular inflammation and increased C4d deposition in the peritubular capillaries of the allograft [47, 51]; thus, a biological relationship exists between anti-HLA DSA strength and the allograft lesion intensity. However, the correlation between the MFI and the antibody level is far from perfect. Despite recent efforts toward the standardization and normalization of solid-phase multiplex-bead arrays [52], there are significant limitations that compromise the use of MFI as a surrogate marker of the antibody level as previously summarized [7, 10, 53, 54]. As a consequence, no consensual threshold for risk categories based on anti-HLA DSA MFI have been defined, a limitation that was pointed out by the Transplantation Society Antibody Consensus Group in 2013 [7]. Recently, Tambur et al. addressed the importance of how best to determine antibody strength and have suggested that the quantification of the antibody level is best achieved by titration [10]. However, the use of anti-HLA DSA titration to predict ABMR and allograft loss has not been incorporated in the routine assessment of anti-HLA DSA and for patient management.

### 3.3. Additional Value of the Complement-Activating Capacity of Donor-Specific Anti-HLA Antibodies for Predicting Outcomes of Kidney Transplantation

Since the pioneering discovery in 1969 that anti-HLA antibodies are lymphocytotoxic [1], activation of the complement cascade has been considered to be a key component of antibody-mediated allograft rejection. However, complement-dependent cytotoxicity assays lack sensitivity and specificity and cannot be used in large scale in transplantation follow-up. The recent development of sensitive solid-phase assays for detecting complement-binding anti-HLA antibodies has revealed novel insights into the associations between anti-HLA DSA and transplant outcomes. Growing evidence supports the notion that the capacity of anti-HLA DSA to bind complement significantly improves our ability to predict ABMR and allograft loss. The clinical relevance of posttransplant complement-binding anti-HLA DSA detected using C1q or C3d assays has been recently shown by several groups in kidney transplantation in the United States and in Europe [18, 20–27] and has also been extended to other solid transplant organs, including heart [19, 30], liver [55], and lung [56]. In the study by Loupy et al. [24], posttransplant C1q-binding anti-HLA DSA detected within the first year after transplantation were found to be an independent determinant of allograft loss with a 4.8-fold increased risk.

Patients with posttransplant C1q-binding anti-HLA DSA exhibited a higher incidence of ABMR and an increased rate of allograft injuries, including microvascular inflammation, tubular and interstitial inflammation, endarteritis, transplant glomerulopathy, and C4d deposition in the peritubular capillaries compared with patients with nonC1q-binding anti-HLA DSA and patients without anti-HLA DSA [24].

Many centers feel that MFI strength is the best predictor of anti-HLA DSA pathogenicity and complement-activating capacity. Recently, anti-HLA DSA complement-binding status following transplantation has been shown to be associated with ABMR occurrence and allograft loss independently of the anti-HLA DSA MFI [24, 57], suggesting an additional value beyond MFI level for outcome prediction. Our team confirmed in a recent prospective study [11] that the detection of complement-binding anti-HLA DSA improved the prediction accuracy for allograft loss at the population level. In this study, the information provided by anti-HLA DSA complement-binding capacity adequately reclassified the individual risk of allograft loss in more than 62% of patients compared with anti-HLA DSA MFI level alone.

### 3.4. The IgG Subclass Composition of Donor-Specific Anti-HLA Antibodies for Predicting Outcomes of Kidney Transplantation

The determinants of anti-HLA DSA complement-binding capacity are complex as discussed previously, including the presence of complement-fixing IgG subclasses (IgG1 and IgG3) and the levels of IgG subclasses [29, 30] (Table 1). Experimental data suggest that antibodies exhibit different abilities to bind complement, to recruit immune effector cells through the Fc receptor, and to display different kinetics of appearance during the immune response according to their IgG1-4 subclass status. [58–60]. Emerging data support the clinical relevance of the IgG subclass composition of anti-HLA DSA and their relationships with allograft injury phenotype and survival in kidney [11, 28, 61–63] and liver [55, 64] transplantation. In particular, several teams have showed a significant association between the IgG3 subclass status of circulating anti-HLA DSA and worse transplant outcome [11, 28, 55, 61, 63, 64].

In a study [28] that included 125 kidney transplant recipients the majority of patients with IgG3 anti-HLA DSA that were detected within the first year after transplantation had acute clinical ABMR that was characterized by intense microvascular inflammation and increased complement deposition in the allografts. In contrast, the majority of patients with IgG4-containing anti-HLA DSA had features of subclinical ABMR with a predominance of chronic features represented by transplant glomerulopathy and interstitial fibrosis. In this study, the IgG3 and IgG4 positivity showed good predictive performance to identify patients with clinical and subclinical ABMR, respectively. Furthermore, it was also shown that circulating anti-HLA DSA IgG3 status improved the performance of MFI level in predicting the individual risk for allograft loss in more than 76% of patients [42].

Overall, in future studies we should evaluate how IgG subtype information may add value to the assessment of sensitized patients and to our current available tools for anti-HLA DSA analysis.

## 4. Risk Stratification Based on Donor-Specific Anti-HLA Antibody Characterization for Transplant Outcome Management

**Figure 2:**
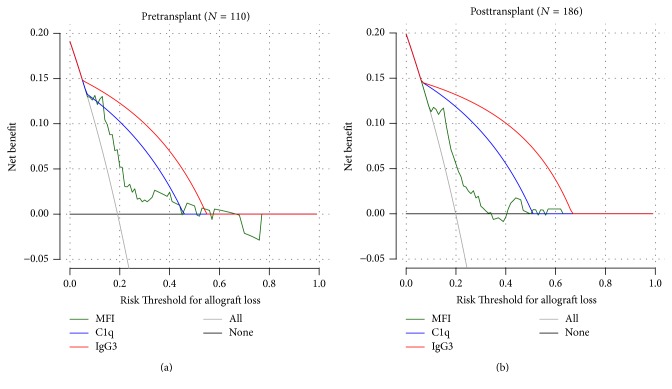
Improvement in clinical decision-making provided by circulating anti-HLA DSA characterization beyond antibody detection: decision curve analysis. Data are based on a prospective study performed in 851 kidney transplant recipients who were screened for the presence of circulating anti-HLA DSA at the time of transplantation, systematically at 1 and 2 years after transplantation, and at the time of any clinical event occurring within the first 2 years after transplantation [11]. Net benefit is shown in the 110 patients identified with pretransplant anti-HLA DSA (a) and in the 186 patients identified with posttransplant anti-HLA DSA (b). Net benefit of a clinical intervention is provided assuming that all patients will lose their graft at 5 years after transplantation (grey) and none of patients will lose their graft at 5 years after transplantation (black), based on anti-HLA DSA MFI level (green), C1q-binding status (blue), and IgG3 subclass status (red). The net benefit is determined by calculating the difference between the expected benefit and the expected harm associated with each decisional strategy. The expected benefit is represented by the number of patients who will lose their allograft and who will undergo clinical intervention (true positives) using the proposed decision rule. The expected harm is represented by the number of patients without allograft loss who would undergo clinical intervention in error (false positives) multiplied by a weighting factor based on the risk threshold. The highest curve at any given risk threshold is the optimal strategy for decision-making in order to maximize net benefit.

The ultimate goal of accurate risk stratification for allograft injury and failure is to improve clinical transplantation outcomes. The risk-stratified approach is greatly needed to tailor therapeutic strategies in the pre- and posttransplant periods, incorporating predicted risks for adverse outcomes to maximize benefits and minimize harms and costs from medical care (Figure 2) [65]. Moreover, risk stratification is also needed to improve our ability to design and interpret therapeutic trials [66]. Averaged results of clinical trials may obscure treatment effect on specific populations, because their aggregated results including patients at various risk levels can be misleading when applied to individual patients [67]. Finally, the risk-stratified approach using anti-HLA DSA properties has direct consequences for patient care. In the pretransplant setting, this approach has the potential to increase allocation policy efficiency by providing more reliable discrimination of the antibodies that are more or less harmful, thereby potentially expanding the donor pool for sensitized patients. In hypersensitized patients with an insufficient stream of potential donors, immunological risk stratification will help to more accurately select the patients in whom specific intensive pretransplant conditioning should be considered to eliminate deleterious antibodies. In the posttransplant setting, systematic monitoring and characterization of circulating anti-HLA antibodies provide a noninvasive tool for clinical decision-making regarding further tests and treatment. In terms of therapeutic strategies, risk assessment based on anti-HLA DSA properties could provide a basis for more targeted pathogenesis-driven therapies. The identification of specific injury phenotypes based on anti-HLA DSA characteristics could provide a rationale for the development of more specific therapeutic approaches, such as B-cell depletion with rituximab in patients with IgG4-associated allograft injury [68] and complement blockade using the C5 inhibitor [69, 70] Eculizumab or C1 inhibitors [71, 72] in patients with complement-binding and/or IgG3-positive anti-HLA DSA. Thus, collaborative prospective analysis of anti-HLA DSA using multiple assays will be critical to reconcile these issues and to create recommendations for best practices.

## Figures and Tables

**Table 1:** Patterns of Class I and Class II HLA specific antibodies in sensitized renal transplant recipients as determined by various modifications of SAB assay (MFI): total IgG, C1q-screen, and IgG1–4 subtypes.

Specificity	Total IgG (MFI)	C1q (MFI)	IgG1 (MFI)	IgG2 (MFI)	IgG3 (MFI)	IgG4 (MFI)
B53	14522	1247	5280	2023	1022	19999
B35	10128	44	2473	178	1516	20667
A23	11440	89	4733	1413	40	0
A2	10605	0	4265	985	475	4
A68	10062	6	29	3463	3	4
B13	8056	1	2763	88	0	0

DR12	11741	30	3864	89	0	5
DR10	19469	6737	8863	1472	0	1551
DQ6	16639	22113	14577	6045	20	9009
DQ7/DQA1*∗*05	16592	7431	14151	5467	21	2811
DQ7/DQA1*∗*03	15287	21936	3901	479	3828	0
DQB1*∗*05:01	16026	20787	14030	5668	0	8066
DR1	10008	3	2388	12	0	0

## References

[1] Patel R., Terasaki P. I. (1969). Significance of the positive crossmatch test in kidney transplantation. *New England Journal of Medicine*.

[2] Nankivell B. J., Kuypers D. R. (2011). Diagnosis and prevention of chronic kidney allograft loss. *The Lancet*.

[3] Sellarés J., de Freitas D. G., Mengel M. (2012). Understanding the causes of kidney transplant failure: the dominant role of antibody-mediated rejection and nonadherence. *American Journal of Transplantation*.

[4] Terasaki P. I. (2003). Humoral theory of transplantation. *American Journal of Transplantation*.

[5] Terasaki P. I. (2012). A personal perspective: 100-year history of the humoral theory of transplantation. *Transplantation*.

[6] Terasaki P. I., Ozawa M. (2004). Predicting kidney graft failure by HLA antibodies: a prospective trial. *American Journal of Transplantation*.

[7] Tait B. D., Süsal C., Gebel H. M. (2013). Consensus guidelines on the testing and clinical management issues associated with HLA and Non-HLA antibodies in transplantation. *Transplantation*.

[8] Djamali A., Kaufman D. B., Ellis T. M., Zhong W., Matas A., Samaniego M. (2014). Diagnosis and management of antibody-mediated rejection: current status and novel approaches. *American Journal of Transplantation*.

[9] Haas M., Sis B., Racusen L. C. (2014). Banff 2013 meeting report: inclusion of C4d-negative antibody-mediated rejection and antibody-associated arterial lesions. *American Journal of Transplantation*.

[10] Tambur A. R., Herrera N. D., Haarberg K. M. K. (2015). Assessing antibody strength: comparison of MFI, C1q, and titer information. *American Journal of Transplantation*.

[11] Viglietti D., Loupy A., Vernerey D. (2016). Value of donor-specific anti-HLA antibody monitoring and characterization for risk stratification of kidney allograft loss. *Journal of the American Society of Nephrology*.

[12] Konvalinka A., Tinckam K. (2015). Utility of HLA antibody testing in kidney transplantation. *Journal of the American Society of Nephrology*.

[13] Schnaidt M., Weinstock C., Jurisic M., Schmid-Horch B., Ender A., Wernet D. (2011). HLA antibody specification using single-antigen beads—a technical solution for the prozone effect. *Transplantation*.

[14] Schwaiger E., Wahrmann M., Bond G., Eskandary F., Böhmig G. A. (2014). Complement component C3 activation: the leading cause of the prozone phenomenon affecting HLA antibody detection on single-antigen beads. *Transplantation*.

[15] Visentin J., Vigata M., Daburon S. (2014). Deciphering complement interference in anti-human leukocyte antigen antibody detection with flow beads assays. *Transplantation*.

[16] Tambur A. R., Glotz D., Herrera N. D. (2016). Can solid phase assays be better utilized to measure efficacy of antibody removal therapies?. *Human Immunology*.

[17] Wahrmann M., Exner M., Regele H. (2003). Flow cytometry based detection of HLA alloantibody mediated classical complement activation. *Journal of Immunological Methods*.

[18] Calp-Inal S., Ajaimy M., Melamed M. L. (2016). The prevalence and clinical significance of C1q-binding donor-specific anti-HLA antibodies early and late after kidney transplantation. *Kidney International*.

[19] Chin C., Chen G., Sequeria F. (2011). Clinical usefulness of a novel C1q assay to detect immunoglobulin G antibodies capable of fixing complement in sensitized pediatric heart transplant patients. *Journal of Heart and Lung Transplantation*.

[20] Comoli P., Cioni M., Tagliamacco A. (2016). Acquisition of C3d-binding activity by de novo donor-specific HLA antibodies correlates with graft loss in nonsensitized pediatric kidney recipients. *American Journal of Transplantation*.

[21] Fichtner A., Süsal C., Höcker B. (2016). Association of C1q-fixing DSA with late graft failure in pediatric renal transplant recipients. *Pediatric Nephrology*.

[22] Freitas M. C. S., Rebellato L. M., Ozawa M. (2013). The role of immunoglobulin-G subclasses and C1q in de novo HLA-DQ donor-specific antibody kidney transplantation outcomes. *Transplantation*.

[23] Guidicelli G., Guerville F., Lepreux S. (2016). Non-complement-binding de novo donor-specific anti-HLA antibodies and kidney allograft survival. *Journal of the American Society of Nephrology*.

[24] Loupy A., Lefaucheur C., Vernerey D. (2013). Complement-binding anti-HLA antibodies and kidney-allograft survival. *New England Journal of Medicine*.

[25] Sicard A., Ducreux S., Rabeyrin M. (2015). Detection of C3d-binding donor-specific anti-HLA antibodies at diagnosis of humoral rejection predicts renal graft loss. *Journal of the American Society of Nephrology*.

[26] Sutherland S. M., Chen G., Sequeira F. A., Lou C. D., Alexander S. R., Tyan D. B. (2012). Complement-fixing donor-specific antibodies identified by a novel C1q assay are associated with allograft loss. *Pediatric Transplantation*.

[27] Yabu J. M., Higgins J. P., Chen G., Sequeira F., Busque S., Tyan D. B. (2011). C1q-fixing human leukocyte antigen antibodies are specific for predicting transplant glomerulopathy and late graft failure after kidney transplantation. *Transplantation*.

[28] Lefaucheur C., Viglietti D., Bentlejewski C. (2016). IgG donor-specific anti-human HLA antibody subclasses and kidney allograft antibody-mediated injury. *Journal of the American Society of Nephrology*.

[29] Schaub S., Hönger G., Koller M. T., Liwski R., Amico P. (2014). Determinants of C1q binding in the single antigen bead assay. *Transplantation*.

[30] Zeevi A., Lunz J., Feingold B. (2013). Persistent strong anti-HLA antibody at high titer is complement binding and associated with increased risk of antibody-mediated rejection in heart transplant recipients. *Journal of Heart and Lung Transplantation*.

[31] Ware J. H. (2006). The limitations of risk factors as prognostic tools. *The New England Journal of Medicine*.

[32] Polak J. F., Pencina M. J., Pencina K. M., O'Donnell C. J., Wolf P. A., D'Agostino R. B. (2011). Carotid-wall intima-media thickness and cardiovascular events. *New England Journal of Medicine*.

[33] Jary M., Vernerey D., Lecomte T. (2015). Prognostic value of angiopoietin-2 for death risk stratification in patients with metastatic colorectal carcinoma. *Cancer Epidemiology Biomarkers and Prevention*.

[34] Pencina M. J., D'Agostino R. B., D'Agostino R. B., Vasan R. S. (2008). Evaluating the added predictive ability of a new marker: from area under the ROC curve to reclassification and beyond. *Statistics in Medicine*.

[35] Pencina M. J., D'Agostino R. B., Steyerberg E. W. (2011). Extensions of net reclassification improvement calculations to measure usefulness of new biomarkers. *Statistics in Medicine*.

[36] Pencina M. J., D'Agostino R. B. (2004). Overall C as a measure of discrimination in survival analysis: model specific population value and confidence interval estimation. *Statistics in Medicine*.

[37] Karpinski M., Rush D., Jeffery J. (2001). Flow cytometric crossmatching in primary renal transplant recipients with a negative anti-human globulin enhanced cytotoxicity crossmatch. *Journal of the American Society of Nephrology*.

[38] Mohan S., Palanisamy A., Tsapepas D. (2012). Donor-specific antibodies adversely affect kidney allograft outcomes. *Journal of the American Society of Nephrology*.

[39] https://optn.transplant.hrsa.gov/governance/policies/

[40] Heidt S., Witvliet M. D., Haasnoot G. W., Claas F. H. J. (2015). The 25th anniversary of the Eurotransplant Acceptable Mismatch program for highly sensitized patients. *Transplant Immunology*.

[41] Süsal C., Roelen D. L., Fischer G. (2013). Algorithms for the determination of unacceptable HLA antigen mismatches in kidney transplant recipients. *Tissue Antigens*.

[42] Wiebe C., Gibson I. W., Blydt-Hansen T. D. (2012). Evolution and clinical pathologic correlations of de novo donor-specific HLA antibody post kidney transplant. *American Journal of Transplantation*.

[43] Haas M., Montgomery R. A., Segev D. L. (2007). Subclinical acute antibody-mediated rejection in positive crossmatch renal allografts. *American Journal of Transplantation*.

[44] Loupy A., Suberbielle-Boissel C., Hill G. S. (2009). Outcome of subclinical antibody-mediated rejection in kidney transplant recipients with preformed donor-specific antibodies. *American Journal of Transplantation*.

[45] Loupy A., Vernerey D., Tinel C. (2015). Subclinical rejection phenotypes at 1 year post-transplant and outcome of kidney allografts. *Journal of the American Society of Nephrology*.

[46] Eskandary F., Bond G., Kozakowski N. (2016). Diagnostic contribution of donor-specific antibody characteristics to uncover late silent antibody-mediated rejection-results of a cross-sectional screening study. *Transplantation*.

[47] Burns J. M., Cornell L. D., Perry D. K. (2008). Alloantibody levels and acute humoral rejection early after positive crossmatch kidney transplantation. *American Journal of Transplantation*.

[48] Archdeacon P., Chan M., Neuland C. (2011). Summary of FDA antibody-mediated rejection workshop. *American Journal of Transplantation*.

[49] Lefaucheur C., Loupy A., Hill G. S. (2010). Preexisting donor-specific HLA antibodies predict outcome in kidney transplantation. *Journal of the American Society of Nephrology*.

[50] Mizutani K., Terasaki P., Hamdani E. (2007). The importance of anti-HLA-specific antibody strength in monitoring kidney transplant patients. *American Journal of Transplantation*.

[51] Papadimitriou J. C., Drachenberg C. B., Ramos E. (2013). Antibody-mediated allograft rejection: morphologic spectrum and serologic correlations in surveillance and for cause biopsies. *Transplantation*.

[52] Reed E. F., Rao P., Zhang Z. (2013). Comprehensive assessment and standardization of solid phase multiplex-bead arrays for the detection of antibodies to HLA. *American Journal of Transplantation*.

[53] Earley M. C., Vogt R. F., Shapiro H. M. (2002). Report from a workshop on multianalyte microsphere assays. *Cytometry*.

[54] Waterboer T., Sehr P., Pawlita M. (2006). Suppression of non-specific binding in serological Luminex assays. *Journal of Immunological Methods*.

[55] O'Leary J. G., Kaneku H., Banuelos N., Jennings L. W., Klintmalm G. B., Terasaki P. I. (2015). Impact of IgG3 subclass and C1q-fixing donor-specific HLA alloantibodies on rejection and survival in liver transplantation. *American Journal of Transplantation*.

[56] Smith J. D., Ibrahim M. W., Newell H. (2014). Pre-transplant donor HLA-specific antibodies: characteristics causing detrimental effects on survival after lung transplantation. *Journal of Heart and Lung Transplantation*.

[57] Bamoulid J., Roodenburg A., Staeck O. (2016). Clinical outcome of patients with de novo C1q-binding donor-specific HLA antibodies after renal transplantation. *Transplantation*.

[58] Nimmerjahn F., Ravetch J. V. (2005). Immunology: divergent immunoglobulin G subclass activity through selective Fc receptor binding. *Science*.

[59] Tao M.-H., Smith R. I. F., Morrison S. L. (1993). Structural features of human immunoglobulin G that determine isotype-specific differences in complement activation. *Journal of Experimental Medicine*.

[60] Van Zelm M. C. (2014). B cells take their time: sequential IgG class switching over the course of an immune response. *Immunology and Cell Biology*.

[61] Everly M. J., Rebellato L. M., Haisch C. E. (2014). Impact of IgM and IgG3 anti-HLA alloantibodies in primary renal allograft recipients. *Transplantation*.

[62] Khovanova N., Daga S., Shaikhina T. (2015). Subclass analysis of donor HLA-specific IgG in antibody-incompatible renal transplantation reveals a significant association of IgG4 with rejection and graft failure. *Transplant International*.

[63] Kimball P., Wagner B., Burton M. (2006). Emergence of IgG3 alloantibody after renal transplantation associated with early graft failure. *Clinical Transplants*.

[64] Kaneku H., O'Leary J. G., Taniguchi M., Susskind B. M., Terasaki P. I., Klintmalm G. B. (2012). Donor-specific human leukocyte antigen antibodies of the immunoglobulin G3 subclass are associated with chronic rejection and graft loss after liver transplantation. *Liver Transplantation*.

[65] Eddy D. M., Adler J., Patterson B., Lucas D., Smith K. A., Morris M. (2011). Individualized guidelines: the potential for increasing quality and reducing costs. *Annals of Internal Medicine*.

[66] Wagner M., Balk E. M., Kent D. M., Kasiske B. L., Ekberg H. (2009). Subgroup analyses in randomized controlled trials: the need for risk stratification in kidney transplantation. *American Journal of Transplantation*.

[67] Kent D. M., Hayward R. A. (2007). Limitations of applying summary results of clinical trials to individual patients: the need for risk stratification. *Journal of the American Medical Association*.

[68] Kamisawa T., Zen Y., Pillai S., Stone J. H. (2015). IgG4-related disease. *The Lancet*.

[69] Cornell L. D., Schinstock C. A., Gandhi M. J., Kremers W. K., Stegall M. D. (2015). Positive crossmatch kidney transplant recipients treated with eculizumab: outcomes beyond 1 year. *American Journal of Transplantation*.

[70] Stegall M. D., Diwan T., Raghavaiah S. (2011). Terminal complement inhibition decreases antibody-mediated rejection in sensitized renal transplant recipients. *American Journal of Transplantation*.

[71] Viglietti D., Gosset C., Loupy A. (2016). C1 inhibitor in acute antibody-mediated rejection nonresponsive to conventional therapy in kidney transplant recipients: a pilot study. *American Journal of Transplantation*.

[72] Montgomery R. A., Orandi B. J., Racusen L. (2016). Plasma-derived C1 esterase inhibitor for acute antibody-mediated rejection following kidney transplantation: results of a randomized double-blind placebo-controlled pilot study. *American Journal of Transplantation*.

